# Mortality due to snakebite and other venomous animals in the Indian state of Bihar: Findings from a representative mortality study

**DOI:** 10.1371/journal.pone.0198900

**Published:** 2018-06-07

**Authors:** Rakhi Dandona, G. Anil Kumar, Archana Kharyal, Sibin George, Md Akbar, Lalit Dandona

**Affiliations:** 1 Public Health Foundation of India, Gurugram, National Capital Region, India; 2 Institute for Health Metrics and Evaluation, University of Washington, Seattle, WA, United States of America; Sciensano, BELGIUM

## Abstract

**Background:**

Animal bites and stings contribute significantly to mortality in certain parts of the world. India accounts for the highest number of snakebites and related mortality globally. We report on mortality due to bite or sting of a venomous animal from a population-based study in the Indian state of Bihar which estimated the causes of death using verbal autopsy.

**Methodology/principal findings:**

Interviews were conducted for all deaths that occurred from January 2012 to March 2014 in 109,689 households (87.1% participation) covering 627,658 population in 1,017 clusters representative of the state using the Population Health Metrics Research Consortium shortened verbal autopsy questionnaire. Cause of death was assigned using the SmartVA automated algorithm. The annualized mortality rate per 100,000 population due to snakebite, scorpion sting and other animals adjusted for age, sex and urban-rural population distribution of the state; and detailed contextual information on snakebites are reported. Deaths due to bite/sting of a venomous animal accounted for 10.7% of all deaths due to unintentional injuries, with an adjusted mortality rate of 6.2 (95% CI 6.0–6.3) per 100,000 population. The adjusted snakebite mortality rate was 4.4 (95% CI 4.3–4.6) which was significantly higher in the rural areas (4.8, 95% CI 4.7–5.0) and in females (5.5, 95% CI 5.3–5.7). Snakebites accounted for 7.6% of all unintentional injury deaths across all ages but for 33.3% of the deaths in 10–14 years age group. A similar proportion of snakebite deaths occurred while sleeping (30.2%), playing (30.2%) and during field/outdoor activities (27.9%). In these cases, 8.2% people were already dead when found, 34.7% had died before treatment could be provided, and 28 (57.1%) had died post treatment among whom 46.4% had sought treatment at a health facility, 25% with a traditional healer, and the rest from both. Death before reaching a health provider, non-availability of medicines or doctor, referral patterns, and sex-differentials in the context of snakebite deaths are reported. None of the verbatim specifically mentioned anti-venom being used for treatment. The adjusted mortality rate for scorpion sting was 0.9 (95% CI 0.8–0.9).

**Conclusions:**

The findings from this large representative sample documents the magnitude of snakebite mortality in Bihar and highlight the circumstances surrounding the snakebite events that could facilitate prevention and intervention opportunities.

## Introduction

Animal bites contribute significantly to morbidity and mortality in children and adults in certain parts of the world, with bites arising from snakes, dogs, cats, and monkeys being the important contributors.[1] Globally, anywhere between 4 to 18 million people are bitten by snakes and 20,000 to 94,000 people die every year with the majority of them in Africa, South-East Asia, and South Asia.[2–4] The wide variations in these estimates are due to scarcity of reliable population-based studies of incidence and mortality.[2–4] Given the extent of the burden of snake bite related mortality and morbidity, the World Health Organization (WHO) recently re-recognised snake bites under the neglected tropical diseases.[5, 6]

India accounts for the highest number of snakebites and related mortality globally.[2] With an age-standardised snakebite mortality rate of 4.1 per 100,000 persons based on a large population-based national survey done nearly 15 years ago, India was estimated to have 46,000 snakebite deaths annually.[7] Three states including Bihar were reported to have the highest annual snakebite deaths.[7] Much of the published literature from India is hospital-based and deals with clinical management of snakebite cases,[8–16] and little information is available around the context around and post snakebite that could facilitate development of successful strategies to address this public health problem.[17]

Using a cluster sampling frame,[18, 19] we conducted a population-based study in Bihar state to estimate the causes of death using verbal autopsy (VA) across all age groups. Bihar state with a population of over 100 million is the third most populous state in India with 11% of it being urban.[20] We report epidemiology of mortality due to bite or sting of a venomous animal for all ages based on VA, and highlight in detail the circumstances surrounding the snakebite events that could facilitate prevention and intervention opportunities.

## Materials and methods

The parent study on causes of death was approved by the Institutional Ethics Committee of the Public Health Foundation of India. All participants provided written informed consent; for those who could not read or write the participant information sheet and consent form were explained by the trained interviewer and a thumb impression obtained.

The sampling method is described in detail elsewhere,[18, 19, 21] and the methods relevant to this report are detailed here. The state of Bihar is divided into 38 districts each of which is divided into 5–27 blocks giving a total of 342 blocks in the state. Within these 342 blocks, the secondary samplings units (SSUs) were villages in rural areas and urban frame survey blocks in urban areas as defined by the National Sample Survey Organization.[22, 23] The SSUs with <50 households were combined with an adjacent SSU, and the large rural SSUs were split into equal sized segments of 100 households using natural boundaries. A total of 1,017 SSUs were sampled in proportion to the number of SSUs in each block, using simple random sampling without replacement. Therefore, this multi-stage stratified random sampling approach to obtain a representative sample of 772 rural and 245 urban clusters provided a total of 1,017 clusters of about 75–150 households across all the 38 districts of the state of Bihar.

This study was conducted from July 2014 to July 2015. In each sampled SSU, all the households (a household was defined as people eating from the same kitchen) were enumerated. During the enumeration, trained interviewers documented the age and sex of all the usual residents in each household of the sampled clusters. Details of members who had in/out-migrated, births, deaths between January 2012 and March 2014 were collected in order to ensure that the denominator included only the usual residents. After documenting the sociodemographic characteristics of the participant, verbal autopsy interviews were conducted for all deaths using the Population Health Metrics Research Consortium (PHMRC) shortened verbal autopsy questionnaires.[24, 25] The respondent for the interview was that household member ≥18 years of age who was most aware of the context of death. A direct question was asked to document if the deceased had suffered an injury/accident that led to the death. Deaths due to bite or sting of a venomous animal were documented under unintentional injury deaths. The animal responsible for bite or sting that resulted in death was documented followed by questions on whether treatment was sought for this bite/sting and if a death certificate for this death was obtained. Following these questions, the respondent was asked to describe the context around the time of bite/sting and death in his/her own words with no prompts from the interviewer as an open verbatim. The PHMRC questionnaire was translated into Hindi (local language), after which it was back-translated into English to ensure the accurate and relevant meaning and intent of the questions. Pilot testing of the questionnaire was carried out and modifications made as necessary. Interview was conducted using the MS-Access and Open Development Kit software in hand-held tablets.

The cause of death was assigned using the validated SmartVA automated algorithm.[26–28] The SmartVA was run on all deaths identified in this population and those due to bite or sting of a venomous animal identified using this run were used in this analysis. We report the annualized incidence estimate of mortality due to venomous animal bite/sting for the state of Bihar using two years data from January 2012 to December 2013 for which population denominator was available. The mortality estimates were adjusted for age, sex and urban-rural distribution of population of Bihar as relevant, and are reported for 100,000 population per year. 95% confidence interval (CI) are reported for all estimates, which was calculated using the formula p ± 1.96 X square root of [p(p-1)/n]; where p is the estimated age-sex rural/urban adjusted rate, n is the number of persons and 1.96 is the approximate value of the 97.5 percentile point of the normal distribution.[29] We also present the distribution of mortality due to venomous animal bite/sting in this population within the unintentional injury mortality, which included road traffic injuries, falls, drowning, fire, and poisoning.

We utilised all cases of venomous animal bites/stings documented from 1 January 2012 to 31 March 2014 for descriptive data analysis. We report on the seasonal trends of venomous animal bite/sting deaths based on the reported date of injury, and the seasons were categorised for Bihar state as per the Indian meteorological department.[30] With snakebites accounting for the largest mortality among these deaths in this population, we present detailed descriptive data for these deaths using the open verbatim with the aim to report the context of snakebite and the patterns that could facilitate understanding of missed opportunities that could guide reduction of mortality due to snakebites. Three team members (AK, SG and MA) reviewed the open verbatim and noted the variety of information available from these reviews including the whether the snake was seen by deceased or someone else, activity the deceased was engaged in at the time of snakebite, what was done immediately after the snakebite, type of treatment received and referral, if any. All cases were reviewed with RD, and modifications made as needed based on re-review of verbatim. We also present case reports across the major themes identified in snakebites to highlight action areas that need attention. Chi-square test is reported where relevant to assess significant associations. Other than SmartVA, the rest of the analysis were performed using STATA 13.0 software (Stata Corp, USA).

## Results

A total of 69 deaths due to bite or sting of a venomous animal were identified from January 2012 to March 2014 (67 deaths in 2012–13) from 109,689 households (87.1% participated, 12.1% door locked, 0.6% migration, and 0.2% other reasons for non-participation) covering 627,658 population of all ages. Verbal autopsy interviews were available for all 69 deaths.

### Mortality due to venomous animal bite

**Fig 1:**
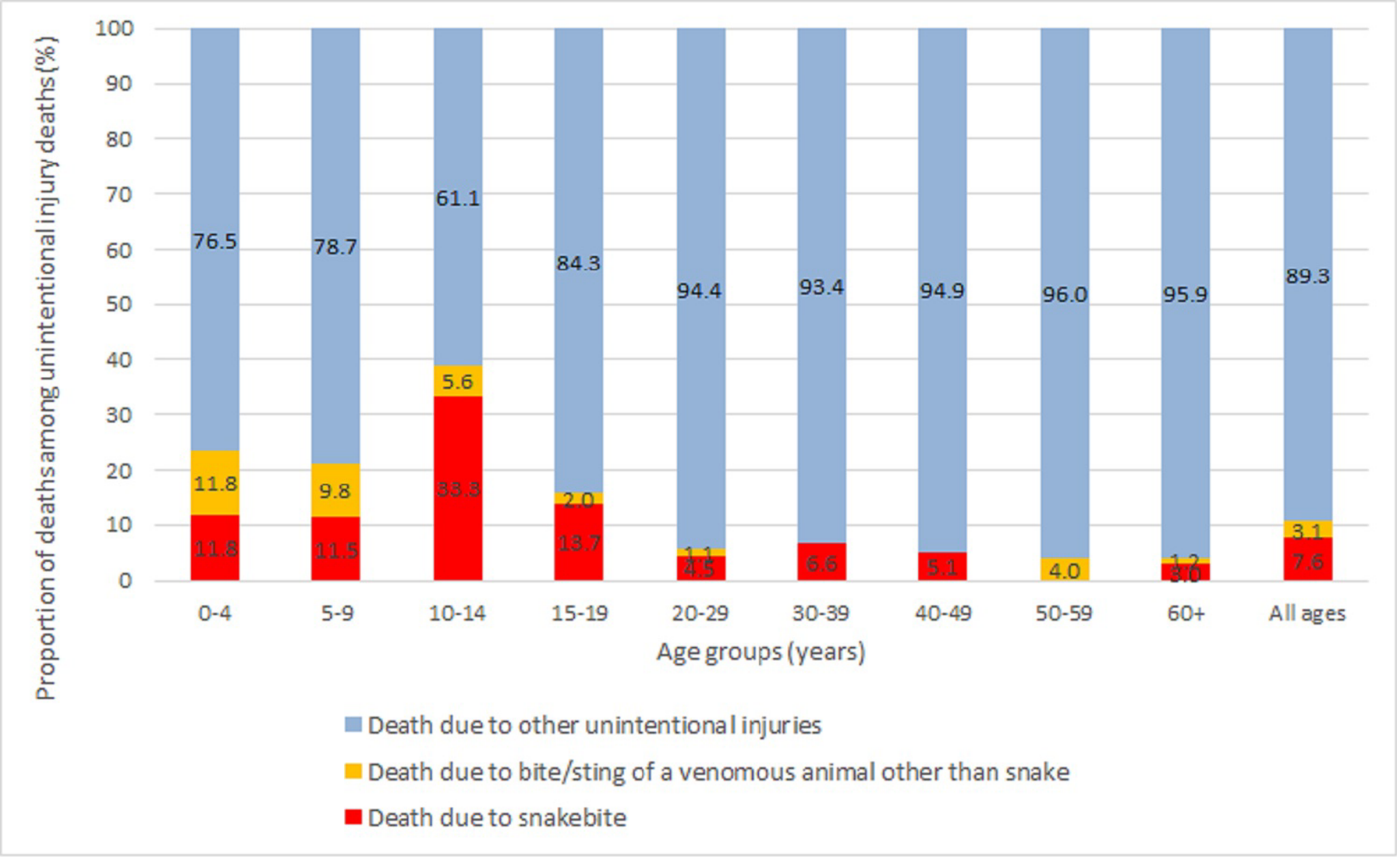
The proportion of deaths due to bite/sting of a venomous animal among the unintentional injury deaths by age group in the Indian state of Bihar using verbal autopsy interviews. Animals other than snake include scorpion, dogs and unknown animal.

The overall adjusted annualised mortality rate due to bite/sting of a venomous animal for all ages was 6.2 (95% CI 6.0–6.3) per 100,000 population, with it being higher in the rural areas than in urban areas (Table 1). The adjusted mortality rate was significantly higher for females (6.9%, 95% CI 6.7–7.2) than for males (5.5%, 95% CI 5.3–5.7) as shown in Table 1. Considering by age, mortality rate was significantly higher in females until 19 years of age than males, but then males had a higher mortality rate in 40–49 years and 60+ years age group (Table 1). Deaths due to bite/sting of a venomous animal accounted for 10.7% of all deaths due to unintentional injuries in this population (Fig 1). It accounted for a significant proportion of unintentional injury deaths in the age groups of 10–14 years (38.9%), 0–4 years (23.5%) and 5–9 years (21.2%). The mean (median) age for mortality due to bite of a venomous animal was 26.7 (20), 15.7 (11), and 20.8 (12) years for males, females, and both sexes combined, respectively. Data on type of animal was available for 65 (94.2%) deaths. Bite by snakes accounted for the majority (49, 71%) followed by scorpion sting (11, 15.9%) and other animal bite including dog and unknown animal (9, 13.1%).

**Table 1:** Annualised adjusted mortality rate due to venomous animal bite/sting based on verbal autopsy interviews for all ages in the Indian state of Bihar. CI denotes confidence interval. Data on type of animal not available for 4 cases.

Variable	Category	Sample		Adjusted mortality rate per 100,000 persons due to venomous animal bite/sting (95% CI)		
		Male	Female	Male	Female	Both
**Age group (years)**	0–4	45,247	42,335	5.3 (4.7–5.8)*	7.8 (7.2–8.5)*	6.5 (6.1–7.0)*†
	5–9	41,666	38,878	7.4 (6.8–8.0)*	11.9 (11.1–12.7)*	9.6 (9.1–10.1)*†
	10–14	39,304	37,594	6.6 (6.1–7.2)*	16.2 (15.2–17.2)*	11.2 (10.6–11.7)*†
	15–19	31,573	28,178	2.6 (2.2–3.1)*	10.2 (9.2–11.1)*	6.0 (5.5–6.4)*†
	20–29	51,069	53,496	2.0 (1.7–2.4)*	1.9 (1.5–2.2)*	1.9 (1.7–2.2)*†
	30–39	39,376	36,098	4.5 (4.0–5.0)*	3.7 (3.2–4.1)*	4.1 (3.8–4.4)*†
	40–49	25,788	22,224	5.1 (4.5–5.8)*	0	2.7 (2.4–3.0)*†
	50–59	18,982	19,663	2.8 (2.2–3.4)*	2.5 (1.9–3.0)*	2.6 (2.2–3.0)*†
	60+	21,370	17,668	14.5 (13.4–15.7)*	3.9 (3.2–4.5)*	9.6 (8.9–10.3)*†
**Place of residence**	Urban	72,480	66,706	2.0 (1.7–2.4)‡	1.5 (1.2–1.8)‡	1.8 (1.5–2.0)†‡
	Rural	241,892	229,426	5.9 (5.7–6.1)‡	7.6 (7.4–7.9)‡	6.7 (6.6–6.9)†‡
**Overall**	All ages	314,371	296,132	5.5 (5.3–5.7)*‡	6.9 (6.7–7.2)*‡	6.2 (6.0–6.3)*†‡

*Urban-rural adjusted;

†Sex-adjusted;

‡Age-adjusted

### Snakebites

**Fig 2:**
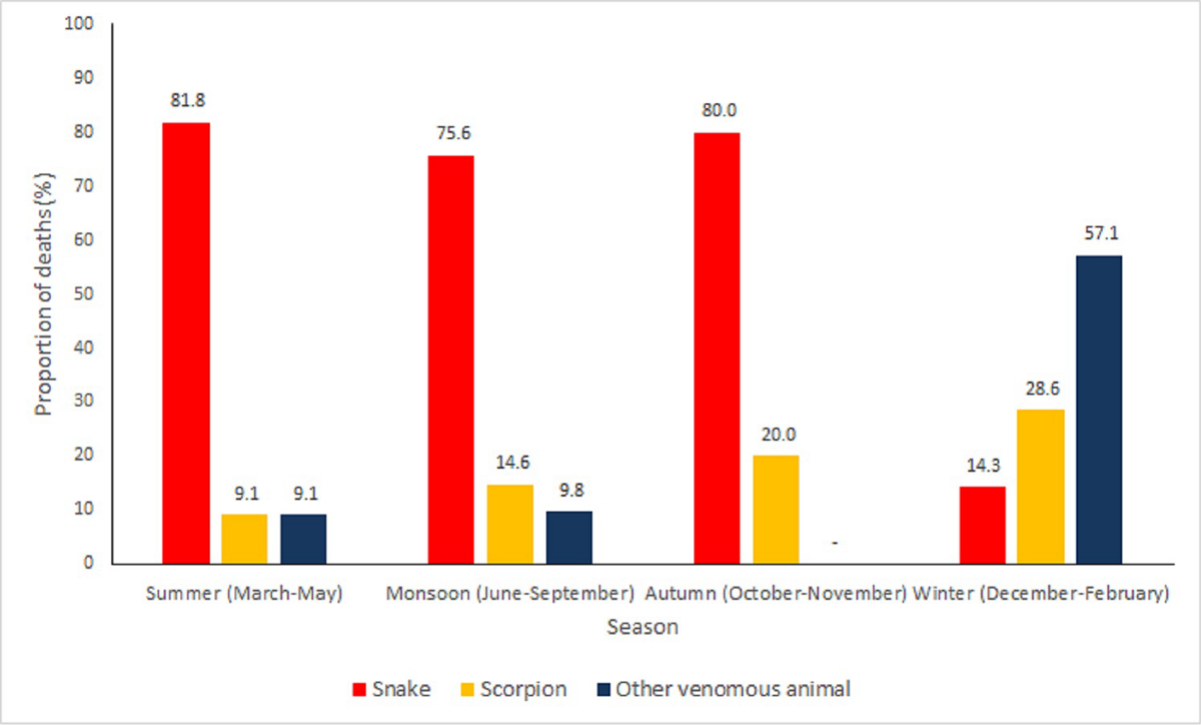
The distribution of deaths due to venomous animal bite/sting by seasonality in the Indian state of Bihar using verbal autopsy interviews.

The overall adjusted annualised snakebite mortality rate was 4.4 (95% CI 4.3–4.6) which was significantly higher in the rural areas (4.8, 95% CI 4.7–5.0) and in females (5.5, 95% CI 5.3–5.7) as shown in Table 2. Though snakebites accounted for 7.6% of all unintentional injury deaths across all ages, however, these accounted for 33.3% of the deaths in 10–14 years age group (Fig 1) with the highest adjusted mortality rate (9.5, 95% CI 9.0–10.1) as shown in Table 2. Age group ≥60 years had the next highest snakebite mortality rate. Considering seasonality of these deaths, snakebite deaths accounted for the most deaths in all seasons except in winter (14.3%, Fig 2).

**Table 2:** Adjusted mortality rate due to venomous animal bite/sting by the type of animal based on verbal autopsy interviews in the Indian state of Bihar. CI denotes confidence interval.

Variable	Category	Adjusted mortality rate per 100,000 persons (95% CI)		
		Snakebite N = 47	Scorpion sting N = 11	Other animal bite N = 9
Age group (years)*†	0–4	3.3 (3.0–3.6)	3.2 (2.9–3.6)	0
	5–9	5.4 (5.0–5.8)	2.6 (2.4–2.9)	1.6 (1.4–1.8)
	10–14	9.5 (9.0–10.1)	0	1.6 (1.4–1.8)
	15–19	5.2 (4.7–5.6)	0.8 (0.6–1.0)	0
	20–29	1.0 (0.8–1.1)	0	0.5 (0.4–0.6)
	30–39	3.2 (2.9–3.5)	0	0
	40–49	1.5 (1.3–1.8)	0	0
	50–59	3.0 (2.5–3.4)	0	2.6 (2.2–3.0)
	> = 60	8.3 (7.6–8.9)	0	3.1 (2.7–3.5)
Sex*‡	Male	3.4 (3.3–3.6)	0.7 (0.7–0.8)	1.2 (1.1–1.3)
	Female	5.5 (5.3–5.7)	1.0 (0.9–1.1)	0.6 (0.5–0.7)
**Place of residence†‡**	Urban	1.4 (1.2–1.6)	0.4 (0.3–0.5)	0
	Rural	4.8 (4.7–5.0)	0.9 (0.9–1.0)	1.0 (1.0–1.1)
Overall*†‡		4.4 (4.3–4.6)	0.9 (0.8–0.9)	0.9 (0.9–1.0)

*Urban-rural adjusted;

†Sex-adjusted;

‡Age-adjusted

Distribution of select characteristics for snakebite deaths based on verbal autopsy interviews by sex are summarised in Table 3. Nearly a similar proportion of snakebite deaths occurred while sleeping (30.2%), playing (30.2%) and during field/outdoor activities (27.9%); females had a higher proportion of snakebites but not statistically significant while sleeping than males (p = 0.925). In 41 (83.7%) of the 49 cases, the snake was seen. In the 8 cases where snake was not seen, doctor informed of the snakebite post medical check-up in 3 cases, fang marks were reported in 1 case, animal bite was mentioned by the deceased to VA respondent in 3 cases, and froth around the mouth and change of skin colour of the deceased to blue or pale was used by the villagers to attribute death to snakebite in 1 case. In these 8 cases, frothing around the mouth was reported in 7 cases and change of skin colour of the deceased in 5 cases. Four (8.2%) people, all females, were already dead when found (Table 3), and the verbatim suggested that snakebite was identified as cause of death based on the froth and/or change in skin colour (Box 1). Seventeen (34.7%) people had died before they could be given any kind of treatment (Table 3). The verbatim of all 17 cases clearly documented that either the deceased died while being taken for treatment or while waiting for the treatment provider to reach the deceased. The case reports in Box 2 also highlight the death occurring between the referral from one provider to another. Overall, 18 (64.3%) of females had died without treatment as compared with 3 (14.3%) of males (p<0.001).

**Box 1. Case reports highlighting the fatal snakebite cases which were found dead by others based on verbal autopsy interviews in the Indian state of Bihar.**

**Case report 1: 14 years/girl, Respondent: Brother**

Brother of the deceased told that his sister had slept at night after dinner. After sometime, someone went to check on her but she was found dead. Her whole body had turned pale and froth was coming from her mouth. Because of the froth, they came to know that a snake had bitten her.

**Case report 2: 18 years/girl, Respondent: Mother-in law**

Respondent told that her daughter-in law was healthy before death. At night, she had prepared and fed food to the family and then slept. When she didn’t wake up in the morning, mother-in law went to wake her up. She saw her mouth was covered with froth and her body was cold to touch and turned pale in colour. The daughter-in-law had already died. Men from the village saw the body and told that snake must have bitten her daughter-in-law.

**Box 2. Case reports highlighting the context of fatal snakebites when the deceased died before treatment could be given based on verbal autopsy interviews in the Indian state of Bihar.**

**Case report 1: 7 months/girl, Respondent: Mother**

Respondent told that her baby was playing in the courtyard around 5 o’clock in the evening when a poisonous snake bit her. Baby was immediately taken to community health centre in Gedawadi but the doctor there told that the baby cannot be treated there. Then, she was taken to Purnia Sadar Hospital, she but died on route to the hospital.

**Case report 2: 4 years/girl, Respondent: Father**

Girl went to a nearby garden to play and wandered into the forest to hide during the play. When she was sitting in the forest, she was bitten by a snake. She went back to other children crying and told them that she was bitten by a snake but they did not believe her and said that she had gotten the cut from wood. The girl went to her mother who suggested to call *jhaadphook* (traditional healer). Her father went to call *jhaadphook* but in the meantime the girl had died.

**Case report 3: 12 years/girl, Respondent: Father**

According to the father of the deceased, the child was grazing an animal in the field. She fell unconscious after a while because of bite of a poisonous animal. Froth was coming from her mouth. They took her home and tried to wake her up after which she vomited twice. Then, a *jhaadphook* (traditional healer) from nearby were called for help but he suggested to take her outside of the village for treatment. Froth was still coming from her mouth, and she died on–route while we were taking her for treatment.

**Table 3:** Distribution of select characteristics for snakebite deaths based on verbal autopsy interviews in the Indian state of Bihar.

	Both (% of N)	Male (% of N)	Female (% of N)
**All snake bites**	**N = 49**	**N = 21**	**N = 28**
Activity at the time of snakebite*‡			
Sleeping	13 (30.2)	3 (17.7)	8 (30.8)
Playing	13 (30.2)	5 (29.4)	10 (38.5)
Field/outdoor activities	12 (27.9)	8 (47.1)	4 (15.4)
Other activities**†**	5 (11.6)	1 (5.9)	4 (15.4)
**Snake seen either by the deceased or someone else‡**			
Yes	41 (83.7)	18 (85.7)	23 (82.1)
No	8 (16.3)	3 (14.3)	5 (17.9)
**Scenario post snakebite**§			
Person was already dead when found	4 (8.2)	-	4 (14.3)
Person died before treatment could be given	17 (34.7)	3 (14.3)	14 (50)
Person died post treatment	28 (57.1)	18 (85.7)	10 (35.7)
**Snakebites with treatment**	**N = 28**	**N = 18**	**N = 10**
**Place of treatment post snakebite‡**			
Health facility only	13 (46.4)	10 (55.6)	3 (30)
Traditional healer only	7 (25)	3 (16.7)	4 (40)
Health facility and traditional healer	8 (28.6)	5 (27.8)	3 (30)
**Referral for treatment‡**			
Yes	6 (21.4)	5 (27.8)	1 (10)
No	22 (78.6)	13 (72.2)	9 (90)

*Data missing for 6 cases

**†**Includes household chores and open defecation

‡Chi-square p-value >0.005 for difference by sex

§Chi-square p-value = 0.002 for difference by sex

Twenty eight (57.1%) people had died post treatment, of which 46.4% had sought treatment at a health facility, 25% with a traditional healer, and the rest from both (Table 3). Though not statistically significant, females were more likely to be seen only by a traditional healer (40%) as compared with males (16.7%, p = 0.172). Six (21.4%) of the 28 cases were referred from one provider to the other. The case reports documented in Box 3 highlight context around the deaths post treatment, including referral and delay in treatment due to non-availability of medicines or doctor. None of the 49 verbatim specifically mention anti-venom being used for treatment; oxygen support for ventilation was mentioned in 2 cases.

**Box 3. Case reports highlighting the context of fatal snakebites when the deceased was given treatment based on verbal autopsy interviews in the Indian state of Bihar.**

**Case report 1: 60 years/female, Respondent: Husband**

Respondent told that on the day of death the deceased was sweeping their courtyard. She stepped on a poisonous snake which bit her. She told the family members that a snake had bitten her and she was immediately taken to a *jhaadphook* (traditional healer) in the village. The healer gave treatment but told that it was difficult for her to survive and within 30 minutes she died.

**Case report 2: 45 years/male, Respondent: Wife**

Respondent narrated the incident that the deceased was cutting leaves of a tree. To cut another branch of that tree, he rested one leg on the wall and the other leg on a brick which was lying below. While stepping down on that brick, a snake bit him. Immediately after the bite his foot was tightly tied and blood was expelled out from the area where snake had bitten. He was taken to Sandhya clinic on a motorcycle where he was admitted. The doctor told that his condition was critical and gave some medicine and saline drip. But there was no improvement in his condition and he died.

**Case report 3: 25 years/male, Respondent: Sister-in law**

According to the respondent the deceased was having a conversation with some people when a snake bit him. He was immediately taken to a *jhaadphook* (traditional healer) but his condition didn’t improve. He was then taken to a hospital opposite the *jhaadphook*’s place. Because medicines were not available in that hospital, he was taken to another hospital but there doctor was not available. So, he was being taken to Gaya for treatment, on-route froth started coming from his mouth and his entire body started to turn light black in colour. The area of bite was full of blood and he then died.

**Case report 4: 65 years/male, Respondent: Wife**

According to wife of the deceased, her husband was tying a bundle of wheat when suddenly a snake bit him. Three people were working with him at that time. They took him to a hospital and drip was given but he was not recovering. His condition was getting worse, so the doctor in this hospital took him to another doctor. But the doctor in that hospital said that he cannot look into this case and he asked him to take the deceased to another hospital. Soon after, froth started coming from his mouth and the finger where the snake had bitten him turned black. He then died.

### Scorpion sting and other animal bites

The overall adjusted annualised scorpion sting and other animal bite mortality rate was 0.9 (95% CI 0.8–0.9) and 0.9 (95% CI 0.9–1.0), respectively (Table 2). The mortality rate with scorpion sting was higher in 0–9 years age group whereas that with the other animal bite was higher in the ≥50 years age group. Half of the scorpion stings occurred while playing, 33.3% while sleeping and the rest while doing other activities. Seven (63.6%) of scorpion sting cases were taken to a health facility and 36.4% cases were taken to a traditional healer immediately post the sting.

## Discussion

The findings from this large representative sample highlights the significant burden of mortality due to snakebites in rural areas, females, and younger ages in the Indian state of Bihar. To our knowledge, these are the first descriptive contextual data from India at population-level that can provide a starting point for possible action and for further in-depth investigations of snakebite mortality.

There was a call to prioritise the snakebite control programme in India following the documentation of the burden of snakebite mortality nationally over 15 years ago.[7] The snakebite mortality rate in our study is similar to that reported for Bihar state over a decade ago,[7] thus suggesting that not much has changed over this period. This unfortunately is true globally as well. Undermining snakebite’s public health importance due to its removal from the neglected tropical disease list by the WHO in year 2013 coupled with anti-venom shortage made it difficult to be dealt with.[5, 6] With snakebite’s reinstatement in the neglected tropical disease list in 2017 and initiation of development of comprehensive snakebite control strategy,[31] there is likely to be an impetus to snakebite prevention and treatment access initiatives.[5] Every year, an estimated 2.8 million people are bitten by snakes in India which makes it imperative for India to prioritise the snakebite control programme on an urgent basis.[3] Calls for including snakebite deaths into the notifiable deaths category in India have gained momentum recently to bring an urgency in managing this public health concern.[32]

Globally, snakebites affect people in rural areas disproportionately.[3, 4, 31, 33] In this study also, snakebite mortality was nearly 3.5 times higher in the rural than in urban areas of the state. Several reasons for a higher rural mortality have been reported such as poorly constructed housing, agricultural work, treatment seeking from traditional healers, and limited access to antivenom.[6] Availability and accessibility of antivenoms is known to be limited in sub-Saharan Africa and Asia,[17, 34, 35] which results in a vicious cycle of poor supply resulting in higher prices which are a deterrent for medical treatment for rural populations, and a lower confidence in the public health sector’s ability to provide effective and safe antivenoms.[6, 36] While considering the treatment seeking behaviour in our study population among those who had died post treatment, only 25% of the deceased were taken to a traditional healer, 46% visited health facility, and 28.6% had sought services both from a health facility and a traditional healer. This distribution possibly reflects less dependence on traditional healers in the studied population. The cases where dual use was documented highlight the non-availability of treatment or non-response to the treatment for considering treatment services from both health facility and a traditional healer. In a recent assessment of treatment seeking behaviour post non-fatal snakebite in Sri Lanka,[37] geographic variation in type of heath care provider sought was noted with allopathic treatment mainly determined by the presence of probable envenoming. Variations in treatment seeking behaviour are seen with traditional healers preferred in Kenya and South Africa,[38, 39] whereas allopathic treatment preferred in Costa Rica.[40] Such data on treatment seeking behaviour for snakebite at population level are not readily available for India. Furthermore, a little over one-third of the snakebite cases in our study population died before they could be provided with any form of treatment, which is similar to that reported from remote areas of Asia and Africa.[33, 41] Two reasons for delay in access to treatment were noted in the open verbatim in our study. First was the delay in knowledge about a snake having bitten the victim to the family members which resulted in delay in accessing treatment. Second was the delay because of refusal/inability of the first point of contact for treatment to provide appropriate treatment. Unfortunately, not even one verbatim categorically mentioned the use of anti-venom in treatment for any deceased. All these findings point to the urgent need of generating data that can facilitate understanding of how people decide where to seek treatment post snakebite and the treatment provided *per se* in order to better understand how to address access to the appropriate treatment in India.[42] Inclusion of snakebite deaths into the notifiable deaths category will allow for an immediate understanding of treatment related issues that need attention for snakebites.[32]

Though there was no significant difference in snakebite mortality by sex nationally in the study over 15 years ago, however, female deaths had exceeded male deaths in 4 states including in Bihar.[7] In our study also, female mortality due to snakebites was 62% higher than that in males. The burden of snakebite mortality was the highest for females in the younger ages, and for males in the older ages. Females in our study were significantly more likely to have died without treatment (both found dead and died before treatment could be given) than males. A higher proportion of snakebites in females while sleeping possibly indicates that they are more likely to sleep on the floor as compared with males who possibly sleep on a bed in rural areas, thereby, increasing exposure to snakebite for females. A common snake in India, the common Krait, bites indoors during night mostly while the victim is asleep who does not wake up at the time of bite because the bite is painless.[43, 44] Sleeping on a bed off the floor in addition to awareness with effective health education and clearing of vegetation have been reported effective in preventing Krait bites.[45] Interestingly, sleeping under a mosquito net, rather than off the floor on a bed, has previously been reported to be protective of snakebite in Nepal.[46] These are simple preventive measures which could be considered in the Indian setting as well to prevent snakebites. Furthermore, with India’s obsession with boy child it is also likely that a girl child is left to play relatively more unsupervised than a boy child, thereby, increasing exposure to mishaps including snakebite. Our contextual data provides pointers to a sex-differential pattern in the snakebite mortality which needs to be understood further within the socio-cultural context of Indian females to prevent and to treat snakebites in females.[47, 48]

For many decades, the "Big 4" snake species have been responsible for Indian snakebite mortality—the common cobra, the common krait, the Russell's viper, and the saw-scaled viper, and there is now the emergence of the hump-nosed pit viper.[49] A recent review highlighted the north-south divide in the snakebite profile in India with neurotoxic envenomations significantly higher in North India compared to South India where hematotoxic envenomations are prevalent.[43] Early morning neuroparalysis caused by common Krait was reported to be a common problem in North India leading to high mortality after snakebite in this review.[43] Documentation of type of snake was beyond the scope of our study, however, the suggestion of less duration between the snakebite and death possibly points to bites by significantly poisonous snakes. Furthermore, with medically important snake species other than the Big 4 being frequently reported from various parts of India,[43] and reports of snakebites being unresponsive to the available polyvalent anti-snake venom which is produced from snakes found in south India,[43, 50] it is imperative that regional variability and snake species other than the Big 4 are also taken into account in the National Snakebite Management Protocol by the government of India and by the WHO South East Asia regional office to prevent this unnecessary mortality.[51, 52]

We found a lower mortality due to bite/sting of other venomous animals as compared with snakes in this population, and most of it was accounted for by scorpion sting. Scorpion envenomation is an important public health hazard in tropical and sub-tropical regions, including India.[53] Annual global number of scorpion stings exceeds 1.2 million, with a higher mortality in children than in adults though adults get more bites.[54] A higher mortality in rural areas following scorpion stings is documented due to lack of access to medical facilities or lack of advances in the treatment of scorpion sting.[53] In the national mortality survey over 15 years ago, the rabies mortality rate was estimated at 1.1 deaths per 100,000 population and Bihar state was among the states with higher mortality rate.[55]

Deaths documented over a two-year period from a large representative well-defined population sample of Bihar, and details of antecedent events and risk factors are the major strengths of this study. Some limitations of these data need to be kept in mind. As this study was designed to capture all causes of death and not specifically the context or risk factors for snakebites *per se*, we have used the information available from the open verbatim to present antecedent events and risk factors. Some of this information maybe subjective to recall bias. However, we believe that the information provided is a useful for action and for further in-depth investigations. Attribution of deaths to snakebite in 3 cases based on history of animal bite coupled with frothing around the mouth and skin colour change of the deceased could also be considered as a limitation. However, given that these signs are quite distinctive for a snakebite,[7, 43, 44, 56] and with a history of animal bite, we believe this attribution to be reasonable. Only in 1 case, this attribution was based on only signs. Also, a reasonably high sensitivity and specificity of verbal autopsy for the snakebite deaths has been documented previously.[7] On the other hand, it is likely that we may have underestimated snakebites in which snake was not seen, particularly when the bite occurred while sleeping at night. The bites by the common Krait, a common snake in India, which mostly bites at night while the victim is asleep,[43, 44] could be missed until the victim reaches a competent doctor who has knowledge of the early morning neuroparalysis caused by common Krait bite.[9, 49]

In conclusion, these data facilitate increasing the visibility of snakebite mortality by addressing many of the gaps in magnitude and risk factors for it in India. These findings also contribute to knowledge around the way forward in the global strategy to combat snake envenoming.[4] Immediate preventive measures such as use of beds and mosquito net can be undertaken immediately by the government to reduce the incidence of snakebite while sleeping. Population level documentation of the incidence of snakebite is needed to fully comprehend the burden of snakebites and treatment seeking behaviour,[37, 57] in addition to mortality to formulate evidence based strategies to address the disproportionate burden of snake envenoming in rural areas and in females in the Indian state of Bihar.

## Supporting information

S1 Checklist STROBE Checklist. (DOC)

## References

[1] World Health Organization. Animal bites: WHO; 2013 [cited 2018 31 January]. Available from: http://www.who.int/mediacentre/factsheets/fs373/en/.

[2] Kasturiratne A, Wickremasinghe AR, de Silva N, Gunawardena NK, Pathmeswaran A, Premaratna R, et al The global burden of snakebite: a literature analysis and modelling based on regional estimates of envenoming and deaths. PLoS medicine. 2008;5(11):e218 Epub 2008/11/07. doi: 10.1371/journal.pmed.0050218 ; PubMed Central PMCID: PMCPMC2577696. 18986210 10.1371/journal.pmed.0050218 PMC2577696

[3] World Health Organization. Prevalence of snakebite envenoming.: WHO; [cited 2018 31 January]. Available from: http://www.who.int/snakebites/epidemiology/en/.

[4] Gutierrez JM, Warrell DA, Williams DJ, Jensen S, Brown N, Calvete JJ, et al The need for full integration of snakebite envenoming within a global strategy to combat the neglected tropical diseases: the way forward. PLoS neglected tropical diseases. 2013;7(6):e2162 Epub 2013/06/21. doi: 10.1371/journal.pntd.0002162 ; PubMed Central PMCID: PMCPMC3681653. 23785526 10.1371/journal.pntd.0002162 PMC3681653

[5] Snake bite—the neglected tropical disease. Lancet (London, England). 2017;390(10089):2 doi: 10.1016/S0140-6736(17)31751-8 . 28677550 10.1016/S0140-6736(17)31751-8

[6] Chippaux JP. Snakebite envenomation turns again into a neglected tropical disease! The journal of venomous animals and toxins including tropical diseases. 2017;23:38 Epub 2017/08/15. doi: 10.1186/s40409-017-0127-6 ; PubMed Central PMCID: PMCPMC5549382. 28804495 10.1186/s40409-017-0127-6 PMC5549382

[7] Mohapatra B, Warrell DA, Suraweera W, Bhatia P, Dhingra N, Jotkar RM, et al Snakebite mortality in India: a nationally representative mortality survey. PLoS neglected tropical diseases. 2011;5(4):e1018 Epub 2011/05/03. doi: 10.1371/journal.pntd.0001018 ; PubMed Central PMCID: PMCPMC3075236. 21532748 10.1371/journal.pntd.0001018 PMC3075236

[8] Ahmed SM, Qureshi UA, Rasool A, Charoo BA, Iqbal Q. Snake bite envenomation in children in Kashmir. Indian pediatrics. 2011;48(1):66–7. Epub 2011/02/15. . 21317471 10.1007/s13312-011-0013-1

[9] Bawaskar HS, Bawaskar PH, Punde DP, Inamdar MK, Dongare RB, Bhoite RR. Profile of snakebite envenoming in rural Maharashtra, India. The Journal of the Association of Physicians of India. 2008;56:88–95. Epub 2008/05/14. . 18472507

[10] Bregani ER, Maraffi T, Tien TV. Snake bites in Moyen Chari district, Chad: a five-year experience. Tropical doctor. 2011;41(2):123–6. Epub 2011/02/10. doi: 10.1258/td.2010.100224 . 21303988 10.1258/td.2010.100224

[11] Chattopadhyay S, Sukul B. A profile of fatal snake bite cases in the Bankura district of West Bengal. Journal of forensic and legal medicine. 2011;18(1):18–20. Epub 2011/01/11. doi: 10.1016/j.jflm.2010.11.007 . 21216374 10.1016/j.jflm.2010.11.007

[12] David S, Matathia S, Christopher S. Mortality predictors of snake bite envenomation in southern India—a ten-year retrospective audit of 533 patients. Journal of medical toxicology: official journal of the American College of Medical Toxicology. 2012;8(2):118–23. Epub 2012/01/12. doi: 10.1007/s13181-011-0204-0 ; PubMed Central PMCID: PMCPMC3550238. 22234395 10.1007/s13181-011-0204-0 PMC3550238

[13] Gautam P, Sharma N, Sharma M, Choudhary S. Clinical and demographic profile of snake envenomation in Himachal Pradesh, India. Indian pediatrics. 2014;51(11):934–5. Epub 2014/11/30. . 25432234

[14] Majumder D, Sinha A, Bhattacharya SK, Ram R, Dasgupta U, Ram A. Epidemiological profile of snake bite in south 24 Parganas district of West Bengal with focus on underreporting of snake bite deaths. Indian journal of public health. 2014;58(1):17–21. Epub 2014/04/22. doi: 10.4103/0019-557X.128158 . 24748352 10.4103/0019-557X.128158

[15] Pore SM, Ramanand SJ, Patil PT, Gore AD, Pawar MP, Gaidhankar SL, et al A retrospective study of use of polyvalent anti-snake venom and risk factors for mortality from snake bite in a tertiary care setting. Indian journal of pharmacology. 2015;47(3):270–4. Epub 2015/06/13. doi: 10.4103/0253-7613.157117 ; PubMed Central PMCID: PMCPMC4450551. 26069363 10.4103/0253-7613.157117 PMC4450551

[16] Punde DP. Management of snake-bite in rural Maharashtra: a 10-year experience. The National medical journal of India. 2005;18(2):71–5. Epub 2005/06/29. . 15981441

[17] Gutierrez JM, Burnouf T, Harrison RA, Calvete JJ, Brown N, Jensen SD, et al A Call for Incorporating Social Research in the Global Struggle against Snakebite. PLoS neglected tropical diseases. 2015;9(9):e0003960 Epub 2015/09/18. doi: 10.1371/journal.pntd.0003960 ; PubMed Central PMCID: PMCPMC4574917. 26379235 10.1371/journal.pntd.0003960 PMC4574917

[18] Kumar GA, Dandona R, Chaman P, Singh P, Dandona L. A population-based study of neonatal mortality and maternal care utilization in the Indian state of Bihar. BMC pregnancy and childbirth. 2014;14:357 Epub 2014/10/19. doi: 10.1186/1471-2393-14-357 ; PubMed Central PMCID: PMCPMC4287469. 25326202 10.1186/1471-2393-14-357 PMC4287469

[19] Kochar PS, Dandona R, Kumar GA, Dandona L. Population-based estimates of still birth, induced abortion and miscarriage in the Indian state of Bihar. BMC pregnancy and childbirth. 2014;14:413 Epub 2014/12/18. doi: 10.1186/s12884-014-0413-z ; PubMed Central PMCID: PMCPMC4300052. 25514837 10.1186/s12884-014-0413-z PMC4300052

[20] Registrar General of India. Population Enumeration Data (Final Population) New Delhi, India2011 [cited 2016 1 June]. Available from: http://www.censusindia.gov.in/2011census/population_enumeration.html.

[21] Dandona R, Kumar GA, Kumar A, Singh P, George S, Akbar M, et al Identification of factors associated with stillbirth in the Indian state of Bihar using verbal autopsy: A population-based study. PLoS medicine. 2017;14(8):e1002363 Epub 2017/08/02. doi: 10.1371/journal.pmed.1002363 . 28763449 10.1371/journal.pmed.1002363 PMC5538635

[22] Registrar General of India. India Population and Housing Census 2001. New Delhi: Office of the Registrar General of India; 2001.

[23] Rahman F, Bose S, Linnan M, Rahman A, Mashreky S, Haaland B, et al Cost-effectiveness of an injury and drowning prevention program in Bangladesh. Pediatrics. 2012;130(6):e1621–8. Epub 2012/11/14. doi: 10.1542/peds.2012-0757 . 23147971 10.1542/peds.2012-0757

[24] Serina P, Riley I, Stewart A, Flaxman AD, Lozano R, Mooney MD, et al A shortened verbal autopsy instrument for use in routine mortality surveillance systems. BMC medicine. 2015;13:302 Epub 2015/12/17. doi: 10.1186/s12916-015-0528-8 ; PubMed Central PMCID: PMCPMC4681088. 26670275 10.1186/s12916-015-0528-8 PMC4681088

[25] Murray CJ, Lopez AD, Black R, Ahuja R, Ali SM, Baqui A, et al Population Health Metrics Research Consortium gold standard verbal autopsy validation study: design, implementation, and development of analysis datasets. Population health metrics. 2011;9:27 Epub 2011/08/06. doi: 10.1186/1478-7954-9-27 ; PubMed Central PMCID: PMCPMC3160920. 21816095 10.1186/1478-7954-9-27 PMC3160920

[26] Serina P, Riley I, Stewart A, James SL, Flaxman AD, Lozano R, et al Improving performance of the Tariff Method for assigning causes of death to verbal autopsies. BMC medicine. 2015;13:291 Epub 2015/12/09. doi: 10.1186/s12916-015-0527-9 ; PubMed Central PMCID: PMCPMC4672473. 26644140 10.1186/s12916-015-0527-9 PMC4672473

[27] Flaxman AD, Vahdatpour A, James SL, Birnbaum JK, Murray CJ. Direct estimation of cause-specific mortality fractions from verbal autopsies: multisite validation study using clinical diagnostic gold standards. Population health metrics. 2011;9:35 Epub 2011/08/06. doi: 10.1186/1478-7954-9-35 ; PubMed Central PMCID: PMCPMC3160928. 21816098 10.1186/1478-7954-9-35 PMC3160928

[28] Flaxman AD, Vahdatpour A, Green S, James SL, Murray CJ. Random forests for verbal autopsy analysis: multisite validation study using clinical diagnostic gold standards. Population health metrics. 2011;9:29 Epub 2011/08/06. doi: 10.1186/1478-7954-9-29 ; PubMed Central PMCID: PMCPMC3160922. 21816105 10.1186/1478-7954-9-29 PMC3160922

[29] WW. L. Confidence intervals for a single mean or proportion: Boston University School of Public Health.; 2016 [cited 2018 21 May]. Available from: http://sphweb.bumc.bu.edu/otlt/MPH-Modules/QuantCore/PH717_ConfidenceIntervals-OneSample/PH717_ConfidenceIntervals-OneSample5.html.

[30] Rathore LS, Attri SD, Jaswal AK. State Level Climate Change Trends in India. 2013.

[31] World Health Organization. Snakebite envenoming: WHO; [cited 2018 31 January]. Available from: http://www.who.int/snakebites/en/.

[32] Shelar J. Make snake bite deaths, cases notifiable: activists to govt.: The Hindu; 2017 [cited 2018 13 April]. Available from: http://www.thehindu.com/news/cities/mumbai/make-snake-bite-deaths-cases-notifiable-activists-to-govt/article19694674.ece.

[33] Chippaux JP. Snake-bites: appraisal of the global situation. Bulletin of the World Health Organization. 1998;76(5):515–24. Epub 1998/12/30. ; PubMed Central PMCID: PMCPMC2305789. 9868843 PMC2305789

[34] Williams D, Gutierrez JM, Harrison R, Warrell DA, White J, Winkel KD, et al The Global Snake Bite Initiative: an antidote for snake bite. Lancet (London, England). 2010;375(9708):89–91. Epub 2010/01/30. doi: 10.1016/s0140-6736(09)61159-4 . 20109867 10.1016/S0140-6736(09)61159-4

[35] Chippaux JP, Massougbodji A, Diouf A, Balde CM, Boyer LV. Snake bites and antivenom shortage in Africa. Lancet (London, England). 2015;386(10010):2252–3. Epub 2015/12/19. doi: 10.1016/s0140-6736(15)01104-6 . 26681284 10.1016/S0140-6736(15)01104-6

[36] Brown NI. Consequences of neglect: analysis of the sub-Saharan African snake antivenom market and the global context. PLoS neglected tropical diseases. 2012;6(6):e1670 Epub 2012/06/09. doi: 10.1371/journal.pntd.0001670 ; PubMed Central PMCID: PMCPMC3367979. 22679521 10.1371/journal.pntd.0001670 PMC3367979

[37] Ediriweera DS KA, Pathmeswaran A, Gunawardena NK, Jayamanne SF, Lalloo DG, de Silva HJ,. Health seeking behavior following snakebites in Sri Lanka: Results of an island. PLoS neglected tropical diseases. 2017;11(11). 10.1371/journal.pntd.0006073 PMC5697880 29108023

[38] Sloan DJ DM, Lalloo DG,. Healthcare-seeking behaviour and use of traditional healers after snakebite in. Trop Med Int Health. 2007;12(11):1386–90. doi: 10.1111/j.1365-3156.2007.01924.x 18045265 10.1111/j.1365-3156.2007.01924.x

[39] Snow RW BR, Roques T, Nyamawi C, Murphy S, Marsh K,. The prevalence and morbidity of snake bite and treatment-seeking behaviour among. Ann Trop Med Parasitol. 1994;88(6):665–71. 7893182 10.1080/00034983.1994.11812919

[40] Arroyo O RG, Gutı ´errez JM. Envenamiento por mordedura de serpiente en Costa Rica en 1996: Epidemiologı ´a y consideraciones clı ´nicas. Acta Me ´dica Costarricense. 1999:23–9.

[41] Fox S, Rathuwithana AC, Kasturiratne A, Lalloo DG, de Silva HJ. Underestimation of snakebite mortality by hospital statistics in the Monaragala District of Sri Lanka. Transactions of the Royal Society of Tropical Medicine and Hygiene. 2006;100(7):693–5. Epub 2005/11/18. doi: 10.1016/j.trstmh.2005.09.003 . 16289649 10.1016/j.trstmh.2005.09.003

[42] Vaiyapuri S, Vaiyapuri R, Ashokan R, Ramasamy K, Nattamaisundar K, Jeyaraj A, et al Snakebite and its socio-economic impact on the rural population of Tamil Nadu, India. PloS one. 2013;8(11):e80090 Epub 2013/11/28. doi: 10.1371/journal.pone.0080090 ; PubMed Central PMCID: PMCPMC3836953. 24278244 10.1371/journal.pone.0080090 PMC3836953

[43] Chauhan V, Thakur S. The North-South divide in snake bite envenomation in India. Journal of emergencies, trauma, and shock. 2016;9(4):151–4. Epub 2016/12/03. doi: 10.4103/0974-2700.193350 ; PubMed Central PMCID: PMCPMC5113082. 27904261 10.4103/0974-2700.193350 PMC5113082

[44] Sharma R, Dogra V, Sharma G, Chauhan V. Mass awareness regarding snake bite induced early morning neuroparalysis can prevent many deaths in North India. International journal of critical illness and injury science. 2016;6(3):115–8. Epub 2016/10/11. doi: 10.4103/2229-5151.190652 ; PubMed Central PMCID: PMCPMC5051053. 27722112 10.4103/2229-5151.190652 PMC5051053

[45] Rodrigo C KS, Gnanathasan A,. Prevention of krait bites by sleeping above ground: preliminary results from an. J Occup Med Toxicol. 2017;12(10):017–0156. 10.1186/s12995-017-0156-7 PMC5368888 28352289

[46] Chappuis F SS, Jha N, Loutan L, Bovier PA,. Protection against snake bites by sleeping under a bed net in southeastern Nepal. The American journal of tropical medicine and hygiene. 2007;77(1):197–9. 17620654

[47] Rao GP, Vidya KL, Sriramya V. The Indian "girl" psychology: A perspective. Indian journal of psychiatry. 2015;57(Suppl 2):S212–5. Epub 2015/09/04. doi: 10.4103/0019-5545.161480 ; PubMed Central PMCID: PMCPMC4539864. 26330637 10.4103/0019-5545.161480 PMC4539864

[48] Razvi M RG. Socio-economic development and gender inequality in India. [cited 2018 18 February]. Available from: https://files.eric.ed.gov/fulltext/ED492144.pdf.

[49] Simpson ID, Norris RL. Snakes of medical importance in India: is the concept of the "Big 4" still relevant and useful? Wilderness & environmental medicine. 2007;18(1):2–9. Epub 2007/04/24. . 17447706 10.1580/06-weme-co-023r1.1

[50] Warrell DA, Gutierrez JM, Calvete JJ, Williams D. New approaches & technologies of venomics to meet the challenge of human envenoming by snakebites in India. The Indian journal of medical research. 2013;138:38–59. Epub 2013/09/24. ; PubMed Central PMCID: PMCPMC3767246. 24056555 PMC3767246

[51] Ministry of Health & Family Welfare GoI. Management of snakebite: quick reference guide New Delhi: GOI; 2016 [cited 2018 13 February]. Available from: http://nhm.gov.in/images/pdf/guidelines/nrhm-guidelines/stg/Snakebite_QRG.pdf.

[52] Warrell DA. Guidelines for the management of snake-bites. India: World Health Organization; 2010 [cited 2018 13 February]. Available from: http://apps.searo.who.int/PDS_DOCS/B4508.pdf.

[53] Bawaskar HS, Bawaskar PH. Scorpion sting: update. The Journal of the Association of Physicians of India. 2012;60:46–55. Epub 2012/06/22. . 22715546

[54] Chippaux JP, Goyffon M. Epidemiology of scorpionism: a global appraisal. Acta tropica. 2008;107(2):71–9. Epub 2008/06/27. doi: 10.1016/j.actatropica.2008.05.021 . 18579104 10.1016/j.actatropica.2008.05.021

[55] Suraweera W, Morris SK, Kumar R, Warrell DA, Warrell MJ, Jha P. Deaths from symptomatically identifiable furious rabies in India: a nationally representative mortality survey. PLoS neglected tropical diseases. 2012;6(10):e1847 Epub 2012/10/12. doi: 10.1371/journal.pntd.0001847 ; PubMed Central PMCID: PMCPMC3464588. 23056661 10.1371/journal.pntd.0001847 PMC3464588

[56] Ariaratnam CA SM, Theakston RD, Warrell DA,. Distinctive epidemiologic and clinical features of common krait (Bungarus caeruleus) bites in Sri Lanka. The American journal of tropical medicine and hygiene. 2008;79(3):458–62. 18784244

[57] Rahman R FM, Selim S, Rahman B, Basher A, Jones A, d'Este C, et al Annual incidence of snake bite in rural bangladesh. PLoS neglected tropical diseases. 2010;4(10):0000860. 10.1371/journal.pntd.0000860 PMC2964284 21049056

